# Nuclear transport receptor importin 11 oppositely regulates viral and bacterial diseases in *Nicotiana benthamiana*

**DOI:** 10.1093/plphys/kiag361

**Published:** 2026-07-13

**Authors:** Xinjian Zhuang, Tingting Li, Ziwei Zhao, Jingquan Tan, Xinying Du, Yuanming Zhang, Yanglin Qiu, Zhongsai Tian, Kai Xu

**Affiliations:** State Key Laboratory of Microbial Technology, Jiangsu Key Laboratory for Pathogens and Ecosystems, Jiangsu Engineering and Technology Research Center for Microbiology, College of Life Sciences, Nanjing Normal University, Nanjing 210023, China; State Key Laboratory of Microbial Technology, Jiangsu Key Laboratory for Pathogens and Ecosystems, Jiangsu Engineering and Technology Research Center for Microbiology, College of Life Sciences, Nanjing Normal University, Nanjing 210023, China; State Key Laboratory of Microbial Technology, Jiangsu Key Laboratory for Pathogens and Ecosystems, Jiangsu Engineering and Technology Research Center for Microbiology, College of Life Sciences, Nanjing Normal University, Nanjing 210023, China; State Key Laboratory of Microbial Technology, Jiangsu Key Laboratory for Pathogens and Ecosystems, Jiangsu Engineering and Technology Research Center for Microbiology, College of Life Sciences, Nanjing Normal University, Nanjing 210023, China; State Key Laboratory of Microbial Technology, Jiangsu Key Laboratory for Pathogens and Ecosystems, Jiangsu Engineering and Technology Research Center for Microbiology, College of Life Sciences, Nanjing Normal University, Nanjing 210023, China; State Key Laboratory of Microbial Technology, Jiangsu Key Laboratory for Pathogens and Ecosystems, Jiangsu Engineering and Technology Research Center for Microbiology, College of Life Sciences, Nanjing Normal University, Nanjing 210023, China; State Key Laboratory of Microbial Technology, Jiangsu Key Laboratory for Pathogens and Ecosystems, Jiangsu Engineering and Technology Research Center for Microbiology, College of Life Sciences, Nanjing Normal University, Nanjing 210023, China; State Key Laboratory of Microbial Technology, Jiangsu Key Laboratory for Pathogens and Ecosystems, Jiangsu Engineering and Technology Research Center for Microbiology, College of Life Sciences, Nanjing Normal University, Nanjing 210023, China; State Key Laboratory of Microbial Technology, Jiangsu Key Laboratory for Pathogens and Ecosystems, Jiangsu Engineering and Technology Research Center for Microbiology, College of Life Sciences, Nanjing Normal University, Nanjing 210023, China

Dear Editor,

Nucleocytoplasmic trafficking is integral to plant immune signaling and pathogen accommodation (Rivas 2012; Castel and Chae 2021), yet the roles of individual nuclear transport receptors (NTRs) in viral infection remain insufficiently defined. We report here that the karyopherin-β import receptor importin 11 (IPO11, also known as KA120) exerts opposing functions in immunity against viral and bacterial pathogens in *Nicotiana benthamiana*. Using stable RNAi knockdown lines together with tobacco rattle virus (TRV)–induced gene silencing, we show that reducing *NbIPO11* enhances systemic infection and accumulation of multiple unrelated positive-stranded RNA viruses and a DNA virus, whereas the same *NbIPO11* knockdown restricts growth of *Pseudomonas syringae* pv. tomato DC3000 (*Pst*DC3000). These findings identify IPO11 as a broadly acting antiviral factor while simultaneously promoting bacterial disease, underscoring a potential tradeoff when manipulating nuclear transport to engineer resistance.

NTRs control the nucleocytoplasmic distribution of immune receptors, transcriptional regulators, and RNA metabolism factors, thereby influencing defense outputs (Rivas 2012; Cui et al. 2016; Jia et al. 2021; Xu et al. 2021; Jia et al. 2023). Viruses likewise depend on host transport pathways, either by encoding nuclear-targeted effectors or by co-opting host trafficking to support replication (Sun et al. 2025), movement (Pascal et al. 1994; Sanderfoot and Lazarowitz 1995), and defense suppression (Kim et al. 2022). Recent work has revealed unexpected specificity within the karyopherin-β family. In *Arabidopsis*, KA120/IPO11 restrains autoimmunity by limiting nuclear activity of the Toll-like/interleukin-1 receptor (TIR) domain-containing nucleotide-binding and leucine-rich repeat (NLR) protein SUPPRESSOR OF NPR1-1, CONSTITUTIVE 1 (SNC1) (Jia et al. 2021) and by suppressing formation of MAC3 (MOS4-ASSOCIATED COMPLEX 3)-dependent nuclear condensates (MDNCs) involving the E3 ligase MAC3B/Pre-mRNA Processing Factor 19 (Prp19) (Jia et al. 2023). In that framework, KA120 acts as a “gatekeeper” that dampens immune activation, and its overexpression can enhance susceptibility to *P. syringae* (Jia et al. 2023) or the powdery mildew pathogen *Golovinomyces cichoracearum* (Jia et al. 2021). Whether IPO11 similarly regulates viral infection has remained unclear, raising the question of whether KA120/IPO11 represents a general immune suppressor or instead produces pathogen-type-specific outcomes.

We first asked how IPO11 relates evolutionarily to other plant NTRs. A neighbor-joining phylogeny built from *Arabidopsis* importin-α, karyopherin-β, and IPO11/KA120 proteins from other plant species revealed that IPO11/KA120 forms an independent, conserved lineage distinct from canonical importin-α and other karyopherin-β clades (Fig. 1a). IPO11 orthologues from diverse plant species, including *N. benthamiana*, clustered with *Arabidopsis* KA120, and NbIPO11 shares high sequence conservation and similar domain architecture with KA120 (Fig. S1). Like *Arabidopsis* KA120 (Jia et al. 2023), NbIPO11 distributes to both the nucleus and the cytoplasm, with predominant nuclear localization (Fig. S2). These relationships support that IPO11 constitutes a conserved karyopherin lineage.

**Figure 1:**
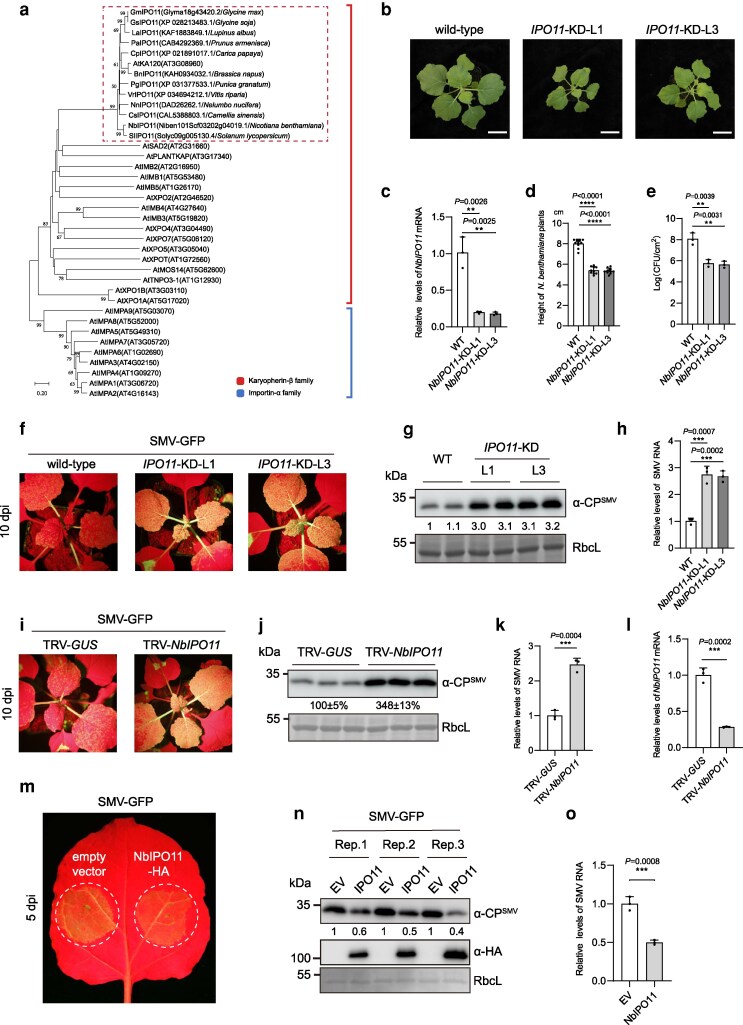
NbIPO11/KA120 is a conserved karyopherin that promotes bacterial susceptibility but restricts SMV infection in *N. benthamiana*. a) Neighbor-joining phylogenetic tree constructed using amino-acid sequences of *Arabidopsis* importin-α and karyopherin-β family members, together with IPO11/KA120 orthologs from 13 plant species, including AtKA120 from *Arabidopsis* and NbIPO11 from *N. benthamiana*. The clades formed by IPO11/KA120 orthologs are boxed with dashed lines. The Bootstrap values with frequencies higher than 50% are shown at nodes; the scale bar indicates amino-acid substitutions per site. b) Representative phenotype of 4-wk-old wild-type (WT) and stable *NbIPO11* knockdown lines (*NbIPO11*-KD-L1 and *NbIPO11*-KD-L3). Scale bar, 5 cm. c) Reverse transcription quantitative PCR (RT-qPCR) confirmation of reduced *NbIPO11* transcript abundance in *NbIPO11*-KD-L1 and *NbIPO11*-KD-L3 relative to WT. d) Plant height quantification for WT and *NbIPO11*-KD lines (*n* = 10). e) Growth of *Pst*DC3000 in WT and *NbIPO11*-KD leaves. Four-wk-old plants were infiltrated with *Pst*DC3000 (OD_600_ = 0.002), and bacterial populations were determined as log (colony-forming unit [CFU]/cm^2^) 2 d after inoculation (*n* = 3). f) Systemic SMV-GFP infection phenotypes in WT and *NbIPO11*-KD lines photographed under UV illumination at 10 days post inoculation (dpi). g) Immunoblot detection of SMV coat protein (CP) in upper leaves from experiments shown in f) using SMV CP–specific polyclonal antibodies; Rubisco large subunit (RbcL) serves as a loading control. Numbers indicate relative band intensities (normalized to WT). h) RT-qPCR quantification of SMV RNA in upper leaves from experiments shown in f) (*n* = 3). i) SMV-GFP systemic infection in TRV-*GUS* (control) and TRV-*NbIPO11* (VIGS) plants photographed under UV illumination at 10 dpi. j) Immunoblot detection of SMV CP in upper leaves from experiments shown in i) with RbcL as loading control; values denote relative CP accumulation. RT-qPCR analysis of SMV RNA k) and *NbIPO11* mRNA l) in upper leaves from experiments shown in i) (*n* = 3). m) Local SMV-GFP fluorescence at 5 dpi in leaves coexpressing empty vector (EV) or NbIPO11 with a hemagglutinin (HA) tag at C-terminus (NbIPO11-HA). n) Immunoblot analysis of SMV CP and NbIPO11-HA in experiments shown in m); RbcL serves as loading control; numbers indicate relative CP levels (normalized to EV). o) RT-qPCR quantification of SMV RNA from experiments shown in m) (*n* = 3). For all quantifications, data are means ± Sd; 2-sided Student's *t*-tests were used, and significance is indicated by asterisks (**P < 0.01; ***P < 0.001; ****P < 0.0001) with *P*-values shown in the graphs. The experiments in f) to o) were repeated 3 times with similar results.

To examine IPO11 function in *N. benthamiana*, we generated stable *NbIPO11* knockdown lines using a hairpin RNAi construct (Supplementary Methods; Table S1) and confirmed substantial depletion of *NbIPO11* transcripts in independent transgenic lines (Fig. 1b to d). As in *Arabidopsis ka120* mutants (Jia et al. 2023), *NbIPO11* knockdown plants exhibited growth defects (Fig. 1b to d), indicating that IPO11 is essential for normal development. Despite these developmental costs, *NbIPO11* knockdown plants supported lower bacterial populations following infiltration with *Pst*DC3000 (Fig. 1e), consistent with *IPO11* acting as a negative regulator of antibacterial immunity and/or a positive contributor to bacterial disease in this system.

In contrast, *NbIPO11* depletion markedly increased infection by a RNA virus, soybean mosaic virus (SMV). In stable *NbIPO11* knockdown lines, the upper leaves infected by a SMV variant expressing green fluorescent protein (GFP) from its genome (SMV-GFP) showed stronger systemic GFP signals and increased viral coat protein and RNA accumulations (Fig. 1f to h). This phenotype was independently reproduced using TRV-induced gene silencing (VIGS) targeting another *NbIPO11* fragment. *NbIPO11*-silenced plants displayed enhanced SMV-GFP systemic infection and increased viral accumulation, concomitant with *NbIPO11* transcript depletion (Fig. 1i to l). Conversely, localized transient overexpression of *NbIPO11* reduced SMV-GFP accumulation in inoculated patches in the same leaf, lowering viral coat protein and viral RNA levels (Fig. 1m to o). Together, these genetic gain- and loss-of-function data identify NbIPO11 as an antiviral restriction factor during SMV infection.

Because *Arabidopsis* KA120/IPO11 has been linked mechanistically to suppression of Prp19/MAC3B nuclear condensates (Jia et al. 2023), we tested whether a similar condensate response accompanies *NbIPO11* depletion or virus infection in *N. benthamiana*. We generated transgenic plants expressing *NbPrp19-GFP* and observed predominant nuclear localization, as expected for Prp19/MAC3 components (Fig. S3). However, NbPrp19-GFP did not form detectable nuclear condensates upon *NbIPO11* knockdown and did not show obvious condensate induction under our SMV (or TRV) infection conditions (Fig. S3). Thus, the IPO11-linked antiviral phenotype in *N. benthamiana* is probably not to be explained by activation of the *Arabidopsis* KA120-MDNC pathway, suggesting mechanistic divergence in downstream wiring despite conservation of the receptor.

We then tested whether NbIPO11-mediated restriction extends beyond SMV. *NbIPO11* knockdown consistently enhanced systemic infection and viral accumulation across a diverse panel of viruses spanning several families, including multiple positive-stranded RNA viruses, such as potyviruses (potato virus Y [PVY], wild tomato mosaic virus [WTMV], clover yellow vein virus [ClYVV]), tobamovirus (tobacco mosaic virus [TMV]), potexvirus (potato virus X [PVX]), alphanecrovirus (tobacco necrosis virus A [TNV-A]), as well as a DNA geminivirus (soybean stay-green associated virus [SoSGV]) (Fig. 2a to g). Importantly, VIGS depletion of *NbIPO11* targeting a different gene fragment produced concordant increases in viral accumulation for these viruses (Fig. S4), supporting that the phenotype reflects reduced IPO11 function rather than off-target effects.

**Figure 2:**
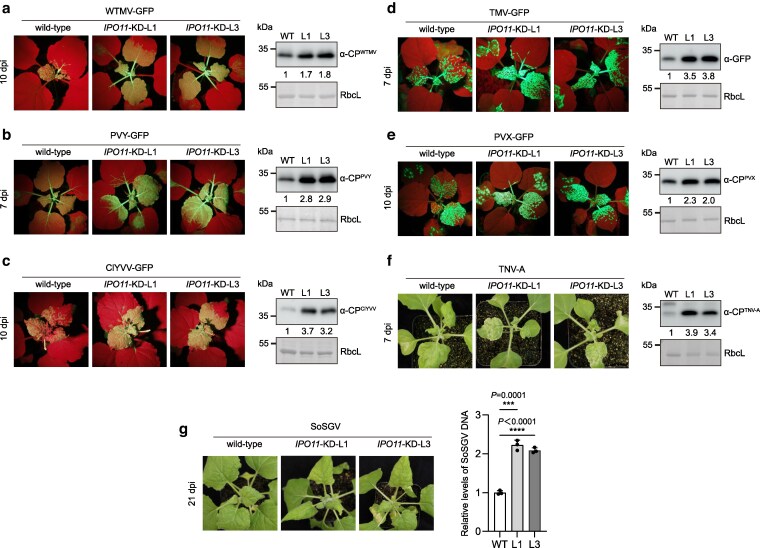
Stable *NbIPO11* knockdown enhances infection by diverse plant viruses. a to e) Representative systemic infection phenotypes of wild-type and *NbIPO11* knockdown lines (*NbIPO11*-KD-L1 and *NbIPO11*-KD-L3) inoculated with viruses expressing GFP from their genomes: WTMV-GFP a), PVY-GFP b), ClYVV-GFP c), TMV-GFP d), and PVX-GFP (e). Plants were photographed under UV illumination to visualize GFP fluorescence in upper/systemic leaves at the indicated time point. Corresponding immunoblots (right) show viral accumulation detected using antibodies against each viral coat protein or GFP (for TMV-GFP); Rubisco large subunit (RbcL) serves as a loading control. The numbers below each band indicate relative viral protein accumulation. f) Representative disease symptoms in wild-type and *NbIPO11*-KD plants inoculated with TNV-A at 7 dpi, with immunoblot detection of TNV-A CP with RbcL as a loading control; numbers denote relative CP levels. g) Representative disease symptoms in wild-type and *NbIPO11*-KD plants inoculated with SoSGV at 21 dpi. qPCR detects the levels of SoSGV DNA in the systemic leaves. Statistical analysis was performed using 2-sided Student's *t*-test. ***P < 0.001; ****P < 0.0001. The experiments in a) to g) were repeated 3 times with similar results.

How can IPO11 promote bacterial disease while restricting viral infection? One parsimonious model is that IPO11 supports a homeostatic module that dampens antibacterial defenses, thereby favoring bacterial proliferation, but simultaneously enabling IPO11-dependent antiviral functions. As a nuclear import receptor, IPO11 could directly deliver antiviral proteins to the nucleus to initiate defense signaling while oppositely regulating the functions of factors involved in antibacterial immunity (Jia et al. 2021). IPO11 may also remodel defense gene expression by controlling the nuclear import of pre-mRNA splicing and mRNA degradation factors; indeed, components of spliceosome, ribonucleoprotein granules, and exosome RNase complexes have been identified as interactors of *Arabidopsis* KA120/IPO11 (Jia et al. 2023). Given that splicing factors regulate plant defense responses (Zhang et al. 2017; Golisz et al. 2021; Du et al. 2024), it remains to be investigated whether IPO11 differentially modulates mRNA processing of defense genes to yield contrasting outcomes in antibacterial versus antiviral responses.

Alternatively, IPO11 could influence the nuclear routing of viral proteins to limit viral replication or spread. However, the breadth of viruses affected by *NbIPO11* knockdown argues against a virus-specific interaction and instead points to a shared host dependency or antiviral pathway controlled by IPO11. Further dissection of IPO11 cargoes and/or determination of how IPO11 remodels defense gene expression are needed to understand the discrepancy in IPO11-regulated nuclear transport between antiviral and antibacterial immunities.

Regardless of detailed mechanisms, while studies of plant immune systems and the underlying cellular regulatory pathways have largely focused on bacterial or fungal pathogens—and have therefore often informed genetic modification strategies aimed at improving resistance to these pathogens—our findings highlight that antiviral defense can be regulated in fundamentally different ways. This divergence is likely to create tradeoffs when immune pathways are manipulated, with distinct consequences for resistance to different pathogen types.

## Supplementary Material

kiag361_Supplementary_Data

## Data Availability

The original contributions presented in the study are included in the article/Supplementary material. Further inquiries can be directed to the corresponding authors.
