# Supplementary material for: Nuclear transport receptor importin 11 oppositely regulates viral and bacterial diseases in Nicotiana benthamiana
Source: Plant Physiol. 2026 Jul 13;201(3):kiag361. doi: 10.1093/plphys/kiag361 (PMC13358392; doi:10.1093/plphys/kiag361)
Supplement: kiag361_Supplementary_Data [file kiag361_supplementary_data.zip › 20260401 Supplementary Figures.pdf]

```
AtKA120   MALSASDLPAMYTLLANSMSGDETVRRPAEAALSLSESRPGFCSCLMEVIASKDLVSHVDVRLMASVYFK   70
NbIPO11   MALSASDLPAMYSLLTNSLSSEQNVRKPAETALAQSENRPGFCSCLMEVITAKDLVSQVDVRLMASVYFK   70
                                                  Importin β N-terminal domain

AtKA120   NSINRHWKSRRNSWSMSNEEKSHLRQKLLSHLREENYQIAEMLAVLISKIARFDYPREWPDLFSVLAQQL  140
NbIPO11   NSINRYWRSRRDSSGISSEEKLHLRQKLLSHLGEENYQIALTLSVIISKIARIDYPKEWPDLFSFLAQQL  140
                           HEAT repeat 1                              HEAT repeat 2

AtKA120   HSADVLASHRIFLILFRTLKELSTKRLTADQKTFAEISSQFFDFSWHLWQTDVQTILHGFSTMVQSYGSN  210
NbIPO11   QSANILTSHRIFMILYRTLKELSTKRLTSDQRTFAEISSQFFDYSWHLWQTDVQTILHGFSALAQTFGGN  210
                                     HEAT repeat 3

AtKA120   SAEQHHDELFLTCERWFLCLKIVRQLIISGFLSDANNIQEIQPVKEVSPALLNAAQSFLPYYSSFQNRDP  280
NbIPO11   AAELNHDDLYLTCERWFLCSKIIRQLIISGFPSDAKTLQEVRHVKEVAPVLLNAIQSLLPYYSSFGDHHP  280
                   HEAT repeat 4                            HEAT repeat 5

AtKA120   KFWEFVKKACVKLMKVLGAIQSRHPFSFGDKCALPVVVDFCLNKITDPEQALLPFEDFFIQCMVMVKSVL  350
NbIPO11   KFWDFLKRACTKLMKILVAIQQRHPYSFGDKCVLPLLMKFCLSKIIDPEPHTMSFEQFMIQCMVMVKTIL  350
                            HEAT repeat 6                           HEAT repeat 7

AtKA120   ECKEYKPSRTGRVMDDNGDTFEQRKKNASNTVGGIVSSLLPNERIVLLCNVLVRRYFVLTASDLEEWYQN  420
NbIPO11   ECKEYKTRLTGRVIDENRVTFEQMKQNISSTVAGLLISILPTDRVVLLCNVLIRRHFVLTASDMEEWYQN  420
                                                   HEAT repeat 8

AtKA120   PESFHHEQDMIQWTEKLRPCAEALYMVLFENYSQLLGPIVVSILQEAMNNCPPSVTEITPALLLKDAAYA  490
NbIPO11   PESFYHEQDSVLWSEKLRPCAEALYIVLFENNGQLLGPVVVSILQEAMSGCPSAVNEITPALLLKDAAYG  490
                            HEAT repeat 9

AtKA120   ATAYVYYELSNYLNFRDWFNGALSLELSNDHPNRRIIHRKVAMILGHWVSEIKDDTKRAVYCALIKLLQD  560
NbIPO11   AAAYIYYELSNYLSFKDWFNGALSLELSNDHPNMRIIHRKVALILGQWVSEIKDDTRRAVYCALIRLLQE  560
                     HEAT repeat 10                           HEAT repeat 11

AtKA120   NDLAVKLAASRSLCLHVEDANFSEQSFLDLLPICWDSCFKMVEVVQEFDSKVQILNLISTLIGHVSEVIP  630
NbIPO11   NDLCVRLTACRSLYFHIEDANFNEKEFLDLLPICWDLCFKLVDEVQEFDSKVQVLNTISVLIARVTEIMP  630
                            HEAT repeat 12                              HEAT repeat 13

AtKA120   YAQKLVQFFQKVWEESSGESLLQIQLLVALRNFVIALGYQSPICYSILLPILQKGIDINSPDSLNLLEDS  700
NbIPO11   YANKLVLFFQKAWEESSSESILQIQLLTALKNFVVALGYQSTKSYRMLLPILQSGININSPD..ELLEDC  698
                                                  HEAT repeat 14

AtKA120   MALWETTLSYAPMMVPQLLALFPYMVEIIERSFDHLQVAVSIMDSYIILDGGEFLNMHASSVAKILDLIV  770
NbIPO11   MQLWEATLINAPSMVPELLGYFPCLVEILERSFDHLKVATNIIEDYVILGGREFLNLHASSVAKLLDLVV  768
                             HEAT repeat 15

AtKA120   GNVNDKGLLSILPVIDILVQCFPVEVPPLISSCLQKLVIICLSGGDDRDPSKTAVKVSSAAILARILVMN  840
NbIPO11   GNVNDKGLLSVIPVIDILVQCFPMEVPQLISSTLQKLIITCLTGGDDHDPSKAAVKASSAALLARILVMN  838
                 HEAT repeat 16                            HEAT repeat 17

AtKA120   TTYLAQLTSDSSLSVLLQQAGVPVEDNILLCLIDIWLDKVDHASPMQQKTFGLALSIILTLRMPQVLDKL  910
NbIPO11   SNYLAQMTSDPSLSIHLQKSGFPSEENILLCLVDIWLEKVDNVTSFQRKTIGLALSIILTLRLPQVLDKL  908
                            HEAT repeat 18                            HEAT repeat 19

AtKA120   DLILSTCTSVILGENKDLTEEESSGDMSSSRSQGEETPPSKELRKSQIKVSDPIYQMSLENSTRENLQTC  980
NbIPO11   DQIMSVCTSVILGGSEDLSEEESSSDSVNS...SKPHVPSKELRRRQMKLSDPVNQISLENSVRDNLQTC  975
                                                   HEAT repeat 20

AtKA120   STLHGDAFNSAISRMHPSALAQVKQALKLP                                        1010
NbIPO11   SALHGESFSAAIGRLHPAVLNQLKQALKMP                                        1005
```

Fig. S1

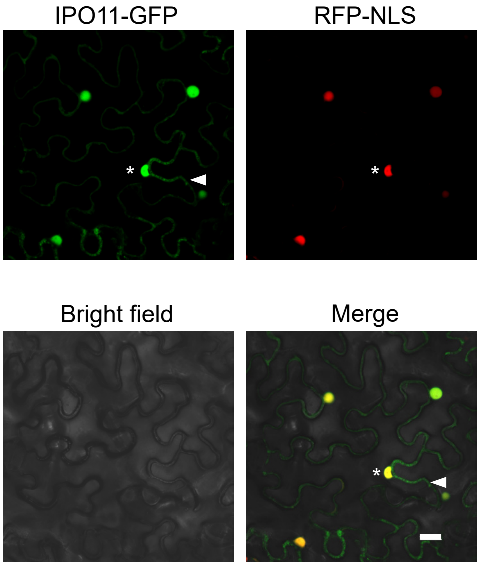

Fig. S2

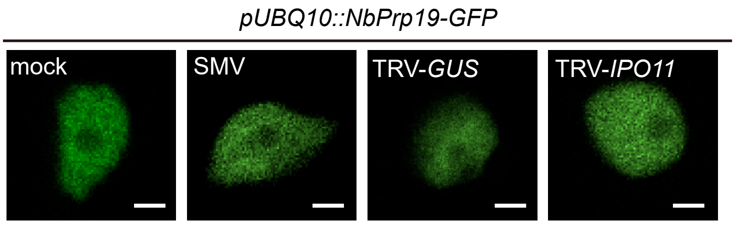

Fig. S3

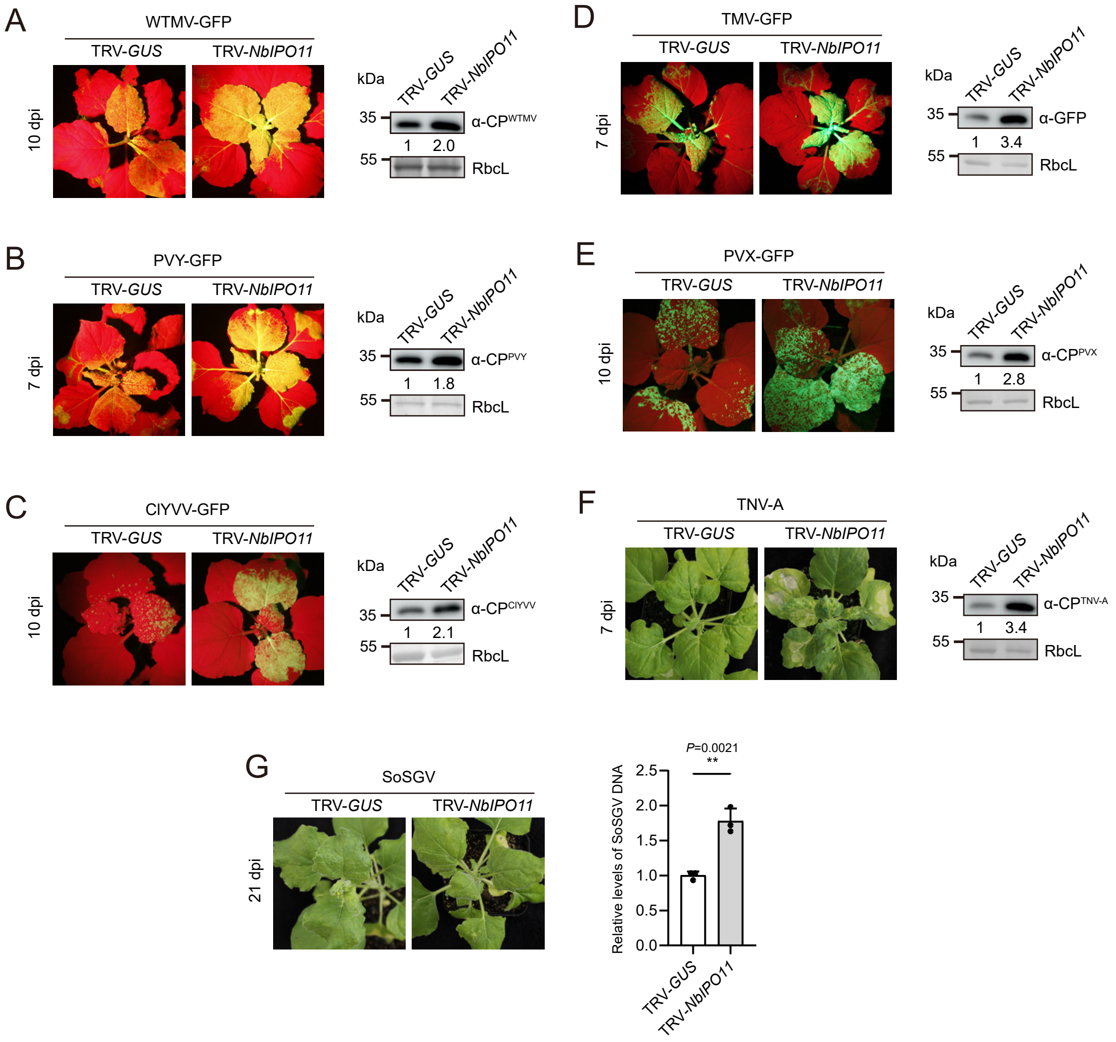

A
WTMV-GFP
TRV-GUS
TRV-NbIPO11
10 dpi
kDa
35
55
α-CP^WTMV
1 2.0
RbcL
B
PVY-GFP
7 dpi
α-CP^PVY
1 1.8
C
ClYVV-GFP
α-CP^ClYVV
1 2.1
D
TMV-GFP
7 dpi
α-GFP
1 3.4
E
PVX-GFP
α-CP^PVX
1 2.8
F
TNV-A
α-CP^TNV-A
1 3.4
G
SoSGV
21 dpi
P=0.0021
**
Relative levels of SoSGV DNA
2.5
2.0
1.5
1.0
0.5
0.0

Fig. S4
