# Supplementary material for: Nuclear transport receptor importin 11 oppositely regulates viral and bacterial diseases in Nicotiana benthamiana
Source: Plant Physiol. 2026 Jul 13;201(3):kiag361. doi: 10.1093/plphys/kiag361 (PMC13358392; doi:10.1093/plphys/kiag361)
Supplement: kiag361_Supplementary_Data [file kiag361_supplementary_data.zip › 20260401 Supplementary Methods.pdf]

Supplementary Methods

Plant materials and growth conditions.

Nicotiana benthamiana plants were cultivated at 25°C under a 16-h-day/8-h-night cycle in a growth chamber.

Plasmids and constructs

The plasmids and corresponding primers used in this study are listed in Table S1. The *NbIPO11* gene sequence was obtained from the Solanaceae Genomics Network (https://www.solgenomics.net). For TRV-mediated gene silencing, the cDNA fragment corresponding to the 1609-1954 nt downstream of the start codon of *NbIPO11* (*Niben101Scf03202g04019.1*) was cloned with primers #4797/#4798. The cDNA fragment was PCR-amplified from *N. benthamiana* cDNA reverse-transcribed using OligoDT and inserted into the pTRV2 vector digested with *Bam*HI/*Xho*I using the Recombinase ClonExpress II One Step Cloning Kit (Cat. No. C112-01, Vazyme, Nanjing, China) to generate pTRV2-*NbIPO11*.

To generate the *NbIPO11*-RNAi vector, a 375-bp fragment (PCR-amplified from cDNA using primers #4799/#4800) was cloned as an inverted repeat into the intron-containing vector pGD-intron2, digested by *BamH*I/*Xho*I and *Bgl*II/*Sal*I. The hairpin structure was subsequently released from the pGD-intron2-based vector by digestion with *Nco*I/*BstE*II, followed by ligation into the *Nco*I/*BstE*II-digested pCambia1301 vector, generating the pCambia1301-*NbIPO11*-RNAi vector for plant transformation.

To construct the vector expressing *NbIPO11-GFP* and *NbPrp19-GFP*, the coding sequence of *NbIPO11* (*Niben101Scf03202g04019.1*) and *NbPrp19* (*Niben101Scf02939g01003.1*) was PCR-amplified using primers #4803/#4804 and #4801/#4802, respectively, and inserted into the vector pGD-C-GFP [1]. The NbPrp19-GFP fragment from the above-mentioned plasmid was subsequently PCR-amplified using primers #5092/#5091 and ligated into the *Nco*I/*BstE*II-digested pCambia1301 vector to generate pCambia1301-NbPrp19-GFP for plant transformation.

To express HA-tagged IPO11, the coding sequence of *NbIPO11* was PCR-amplified using primers #4803/#4804, digested with *BamH*I/*Xho*I, and ligated with vector pGD-C-HA digested by *BamH*I/*Xho*I.

Generation of transgenic *N. benthamiana* plants

The *NbIPO11*-KD and *pUBQ10::NbPrp19-GFP* transgenic *N. benthamiana* plants were generated through *Agrobacterium*-mediated transformation. The leaf disc transformation method was performed to generate the transgenic plants [2]. PCR-positive T0 generation lines of *NbIPO11*-KD transgenic plants were verified by RT-qPCR for successful knockdown. To confirm *pUBQ10::NbPrp19-GFP* transformation into *N. benthamiana*, T0 generation plants were tested by western blot and confocal microscopy imaging to test the expression of *NbPrp19-GFP*. For both transformations, stable T2 generation lines were selected and used for experiments.

Pseudomonas syringae pathovar (pv) tomato DC3000 growth assays

*Pst*DC3000 growth assays were conducted as previously described [3]. Bacterial cultures were harvested and resuspended in 10 mM $MgSO_4$ to an OD_{600} of 0.002. The suspension was pressure-infiltrated into the abaxial leaf surface of 4-week-old plants using a 1-mL needleless syringe. Bacterial growth was assessed at 2 days post-inoculation (dpi). Prior to quantification, leaf surfaces were rinsed with sterile water. For colony counting, three discs from three leaves of a single plant were pooled and homogenized in 750 μL of 10 mM $MgCl_2$. The homogenate was serially diluted and spot-plated onto selective King's B medium (containing per liter: 20 g protease peptone, 1.5 g K_2HPO_4, 1.5 g 1 M $MgSO_4$, 10 g agar, and *Pseudomonas* CFC selective antibiotics). Plates were incubated at 28 °C for 48 h before colonies were counted.

VIGS of *NbIPO11* genes in *N. benthamiana*

A tobacco rattle virus (TRV)-based VIGS system was used to silence the *NbIPO11* gene in *N. benthamiana*. Briefly, 3-week-old *N. benthamiana* plants were agroinfiltrated with an *Agrobacterium* mixture containing pTRV1 (OD_{600} = 0.2) and pTRV2 (OD_{600} = 0.2) vectors. The silenced plants were further inoculated with an *Agrobacterium* harboring infectious clones of SMV-GFP [4], ClYVV-GFP [5], PVY-GFP [6], WTMV-GFP [7], TMV-GFP, PVX-GFP, TNV-A, or SoSGV [5], respectively.

The *NbIPO11* mRNA and viral RNA levels in the systemically infected leaves were accessed by RT-qPCR. All VIGS experiments were repeated 3 times with 5 plants per treatment.

Virus inoculation and fluorescence imaging

Agrobacterium cultures carrying the respective viral infectious clones were resuspended in MMA buffer (10 mM MES, 10 mM $MgCl_2$, and 150 μM acetosyringone, pH 5.6) with an OD_{600} of 0.5 and incubated at room temperature for 3 h. The *Agrobacterium* suspension was infiltrated into the leaves of *N. benthamiana* plants using a needleless syringe. GFP fluorescence in the assayed plant leaves was visualized using a handheld long-wave UV lamp with emitting light at 360–370 nm (LUYOR-3410, Luyor, Shanghai, China) for exciting GFP fluorescence expressed from TMV-GFP and PVX-GFP or a handheld LED lamp with emitting light at 440–460 nm (LUYOR-3415RG, Luyor, Shanghai, China) for exciting EGFP fluorescence expressed from SMV-GFP, WTMV-GFP, PVY-GFP, and ClYVV-GFP. Leaves were photographed using a Canon EOS 1500D digital camera installed with an LP510 long-pass filter.

Protein extraction and immunoblot analysis

Protein extraction and immunoblot analysis methods were performed as described previously [8]. Plant tissues were extracted using SDS loading buffer (4% [w/v] SDS,

5% [v/v] β-mercaptoethanol, 100 mM Tris-HCl, pH 6.8, 20% [v/v] glycerol, and 0.2%[w/v] bromophenol blue). Total proteins were separated in SDS-polyacrylamide gels and then transferred onto polyvinylidene fluoride (PVDF) membranes (Bio-Rad, Cat. #1620177) for western blotting. Proteins were detected using specific primary antibodies: anti-GFP (Sangon Biotech, D191040), anti-HA (Sangon Biotech, D110004), anti-CP^{SMV}, anti-CP^{WTMV}, anti-CP^{ClYVV}, anti-CP^{PVY}, anti-CP^{PVX}, and anti-CP^{TNV} (1:5000 dilution). After incubation with HRP-conjugated secondary antibodies (Sangon Biotech, D110058 or D110097), blots were developed with Chemistar High-sig ECL substrate (Tanon, 180-501) and imaged on an Amersham Imager 680 (GE Healthcare). The Ponceau S-stained Rubisco large subunit (RbcL) protein was used as a loading control.

RNA extraction and reverse transcription-quantitative real-time PCR analysis

RNA extraction and RT-qPCR analysis were performed as previously described [8]. The β-tubulin coding gene was used as an internal control. The results were analyzed by the $2^{-\Delta\Delta Ct}$ method and shown as means ± SD (n = 3). The primers employed for RT-qPCR are listed in Table S1.

DNA extraction and quantitative real-time PCR analysis

DNA from about 20 mg of SoSGV-infected plant tissues was extracted using the CTAB method as previously described [5]. Quantitative real-time PCR analysis was

carried out to analyze relative accumulation of SoSGV genome DNA. The *EF1A* was used as an internal control. The results were analyzed by the 2^-ΔΔCt method and shown as means ± SD (n = 3). The primers employed for qPCR are listed in Table S1.

Subcellular localization assay

To assess nuclear condensate formation of NbPrp19-GFP upon SMV infection, leaves of 4-week-old *pUBQ10::NbPrp19-GFP* transgenic plants were infiltrated with *Agrobacterium* mixture containing SMV vectors (OD_{600} = 0.2). Fluorescence images of NbPrp19-GFP were taken at 5 dpi. To investigate the role of *NbIPO11* in NbPrp19-GFP nuclear condensate formation, systemic leaves from TRV-*IPO11*-silenced *pUBQ10::NbPrp19-GFP* plants were subjected to confocal microscopy, with TRV-*GUS*-infected plants serving as controls. To assess the subcellular localization of NbIPO11, *Agrobacterium* mixture containing expression vectors NbIPO11-GFP (OD_{600} = 0.4), NLS-RFP (OD_{600} = 0.4), and TBSV P19 (OD_{600} = 0.2) were co-infiltrated into *N. benthamiana* leaves. GFP fluorescence was excited with a 488 nm laser and detected at 500–550 nm, while RFP fluorescence was excited with a 561 nm laser and detected at 570–620 nm, using a Nikon A1 confocal laser-scanning microscope (Ti-E-A1R, Nikon, Japan).

Phylogenetic analysis

The importin sequences were obtained from TAIR (https://www.arabidopsis.org),

Solanaceae Genomics Network (https://www.solgenomics.net), or Soybase (https://www.soybase.org). Phylogenetic analysis was performed in MEGA12 (https://www.megasoftware.net/) using the Neighbor-joining method.

1. Xu, K. and P.D. Nagy, *Enrichment of Phosphatidylethanolamine in Viral Replication Compartments via Co-opting the Endosomal Rab5 Small GTPase by a Positive-Strand RNA Virus.* PLoS Biol, 2016. **14**(10): p. e2000128.
2. Latif, M.F., et al., *Transgenic expression of artificial microRNA targeting soybean mosaic virus P1 gene confers virus resistance in plant.* Transgenic Res, 2024. **33**(3): p. 149-157.
3. Ding, Y., et al., *Opposite Roles of Salicylic Acid Receptors NPR1 and NPR3/NPR4 in Transcriptional Regulation of Plant Immunity.* Cell, 2018. **173**(6): p. 1454-1467.e15.
4. Yin, J., et al., *A cell wall-localized NLR confers resistance to Soybean mosaic virus by recognizing viral-encoded cylindrical inclusion protein.* Mol Plant, 2021. **14**(11): p. 1881-1900.
5. Yin, J., et al., *Leafhopper transmits soybean stay-green associated virus to leguminous plants.* Phytopathology Research, 2023. **5**(1): p. 17.
6. Sun, K., et al., *Rapid Construction of Complex Plant RNA Virus Infectious cDNA Clones for Agroinfection Using a Yeast-E. coli-Agrobacterium Shuttle Vector.* Viruses, 2017. **9**(11).
7. Yin, J., et al., *The Characterization of the Tobacco-Derived Wild Tomato Mosaic Virus by Employing Its Infectious DNA Clone.* Biology (Basel), 2022. **11**(10).
8. Zhuang, X., et al., *Rice stripe virus NS3 uses the host signaling pathways to control pathogenicity.* Dev Cell, 2025. **60**(17): p. 2363-2379.e8.
