# Supplementary material for: Nuclear transport receptor importin 11 oppositely regulates viral and bacterial diseases in Nicotiana benthamiana
Source: Plant Physiol. 2026 Jul 13;201(3):kiag361. doi: 10.1093/plphys/kiag361 (PMC13358392; doi:10.1093/plphys/kiag361)
Supplement: kiag361_Supplementary_Data [file kiag361_supplementary_data.zip › figure_legends.docx]

**Fig. 2 Stable *NbIPO11* knockdown enhances infection by diverse plant viruses.** (**A-E**) Representative systemic infection phenotypes of wild-type and *NbIPO11* knockdown lines (*NbIPO11*-KD-L1 and *NbIPO11*-KD-L3) inoculated with viruses expressing GFP from their genomes: wild tomato mosaic virus (WTMV-GFP) (A), potato virus Y (PVY-GFP) (B), clover yellow vein virus (ClYVV-GFP) (C), tobacco mosaic virus (TMV-GFP) (D), and potato virus X (PVX-GFP) (E). Plants were photographed under UV illumination to visualize GFP fluorescence in upper/systemic leaves at the indicated time point. Corresponding immunoblots (right) show viral accumulation detected using antibodies against each viral coat protein or GFP (for TMV-GFP); RbcL serves as a loading control. The numbers below each band indicate relative viral protein accumulation. (**F**) Representative disease symptoms in wild-type and *NbIPO11*-KD plants inoculated with tobacco necrosis virus A (TNV-A) at 7 dpi, with immunoblot detection of TNV-A CP with RbcL as a loading control; numbers denote relative CP levels. (**G**) Representative disease symptoms in wild-type and *NbIPO11*-KD plants inoculated with soybean stay-green associated virus (SoSGV) at 21 dpi. Quantitative PCR detects the levels of SoSGV DNA in the systemic leaves. Statistical analysis was performed using two-sided Student’s *t*-test. ***, *P*<0.001; ****, *P*<0.0001. The experiments in panels A-G were repeated three times with similar results.

**Fig. S1 Amino-acid sequence conservation between *Arabidopsis* KA120 and *Nicotiana benthamiana* IPO11.** Pairwise alignment of AtKA120 and NbIPO11 protein sequences. Identical residues are highlighted in black, and conserved substitutions are highlighted in grey. Numbers on the right indicate amino-acid positions. The Importin β N-terminal domain, responsible for binding to the Ran GTPase, and 20 HEAT repeats are annotated. The extensive conservation across the full-length proteins supports that NbIPO11 is the KA120/IPO11 orthologue in *N. benthamiana*.

**Fig. S2 Subcellular localization of IPO11-GFP in *N. benthamiana*.** Agrobacteria cells carrying expression vectors for IPO11-GFP and RFP-NLS (a nucleus marker) were infiltrated into the leaves of *N. benthamiana* plants. The subcellular localization of IPO11-GFP and RFP-NLS in epidermal cells was observed 3 days after agroinfiltration using a confocal microscopy. An asterisk annotates the nuclear-localized IPO11-GFP; a triangle indicates a minor cytoplasmic distribution.

**Fig. S3 Confocal images of NbPrp19-GFP in the nucleus.** Transgenic *N. benthamiana* plants expressing *NbPrp19-GFP* under the control of *Arabidopsis* *UBP10* promoter (*pUBQ10*::*NbPrp19-GFP*) were either mock-inoculated or inoculated with SMV, TRV-*GUS*, or TRV-*IPO11*. Nuclear NbPrp19-GFP without detectable condensate formation was observed. Scale bar, 2 μm.

**Fig. S4 TRV-induced *NbIPO11* silencing enhances accumulation of multiple plant viruses.** (**A-G**) Representative systemic infection phenotypes and analysis of viral CP or genomic DNA accumulation in TRV-*GUS* (control) and TRV-*NbIPO11*-treated *N. benthamiana* plants inoculated with viruses: WTMV-GFP (A), PVY-GFP (B), ClYVV-GFP (C), TMV-GFP (D), PVX-GFP (E), TNV-A (F), and SoSGV (G). Plants were photographed under UV illumination to visualize the fluorescence of GFP expressed from the viral genome (panels A-E) or under normal light to visualize TNV-A- and SoSGV-induced symptoms (panels F-G) in upper/systemic leaves at the indicated time point. Viral proteins were detected using antibodies against the indicated viral CP or GFP (for TMV-GFP). RbcL serves as a loading control. Numbers below the blots indicate relative viral protein accumulation in TRV-*NbIPO11* plants normalized to TRV-*GUS* (set to 1). SoSGV DNA accumulation in systemic leaves was measured by quantitative PCR. Statistical analysis was performed using two-sided Student’s *t*-test. **, *P*<0.01. The experiments in panels A-G were repeated three times with similar results.
