# Supplementary material for: Nuclear transport receptor importin 11 oppositely regulates viral and bacterial diseases in Nicotiana benthamiana
Source: Plant Physiol. 2026 Jul 13;201(3):kiag361. doi: 10.1093/plphys/kiag361 (PMC13358392; doi:10.1093/plphys/kiag361)
Supplement: kiag361_Supplementary_Data [file kiag361_supplementary_data.zip › Table S1 Primers designed and used in this study.pdf]

Table S1 Sequencing primers designed and used in this study

Primer name	Primer sequence (5’→3’)	Purpose
4799/NbIPO11/BamHI/F	CGCGGATCCGTCGCTATCCAGCAGCGACATCC	pCAMBIA1301-NbIPO11-RNAi, RT-qPCR
4800/NbIPO11/XhoI/R	CCGCTCGAGCTCTGGGTTCTGGTACCATTCCTCC	
4797/ NbIPO11/TRV/BamHI/F	CGCGGATCCCAATGGGTTTCAGAGATTAAAGATGACACC	pTRV2-NbIPO11, RT-qPCR
4798/ NbIPO11/TRV/XhoI/R	CCGCTCGAGATGCTTTCGCTAGAAGATTCTTCCCATG	
4803/NbIPO11/pGD-C/BamHI/F	CATCTAGAACTAGTGGATCCATGGCTCTATCAGCCTCCGACTTACC	pGD-NbIPO11-HA, pGD-NbIPO11-GFP
4804/NbIPO11/pGD-C/EcoRI/R	ATAAGCTTGATATCGAATTCTGGCATCTTCAATGCTTGTTTTAATTGATTG	
1739/SMV/CP/F	TCAGGCAAGGAGAAGGAAGG	RT-qPCR
8//SMV/CP/R	GCTCATATTCATCTTTAACTGCATTGTACCAC	
143/NbTubulin/F	CAGTGTCCTGTCAACTCATTCCCTCCTTG	RT-qPCR reference
144/NbTubulin/R	GTTAATACCACACTTGAATCCAGTAGGGCACC	
4801/NbPrp19/pGD-C/BamHI/F	CATCTAGAACTAGTGGATCCATGAACTGTTCAATTTCCGGCGAGG	pGD-NbPrp19-GFP
4802/NbPrp19/pGD-C/EcoRI/R	ATAAGCTTGATATCGAATTCACTCTCCATTTGATCCTCGCCAGGC	
5092/NbPrp19-GFP/pCAMBIA1301/NcoI/F	GTTTTTCTGATTAACAGCCATGGTGAACTGTTCAATTTCCGGCG	pCAMBIA1301-NbPrp19-GFP
5091/GFP/pCAMBIA1301/BstEII/R	GGAAATTCGAGCTGGTCACCTCACTTGTACAGCTCGTCCATGC	
3665/pGD/XhoI/IntronII/F	TCCGGGCCCTCTAGACTCGAGAGGTAAGTATGCACTTAAAGAGTATGTG	pGD-intron2
3666/pGD/AvrII/IntronII/R	TCTCTGCAGGTCGACCCTAGGACCTGCATAATTTCAAAGATTGAACC	
3792/pGD/IntronII/lengthen/BamHI/F	GCCGGATCCGGGCCCTCTAGACTCG	
3793/pGD/IntronII/lengthen/SacI/R	GAACGAGCTCGGTCACCAGATCTCTGCAGGTCGACCCTAG	
5182/pGD-TEV-leader/-NcoI-BamHI/R	CGCGGATCCCCATGGGGCGCGCCGATCCGGCTATCGTTC	
851/HindIII/3xHA/BglII/F	AGCTTTATCCATACGATGTTCCAGATTATGCTGGTTACCCTTATGATGTTCCTG ATTACGCTGGCTATCCATACGATGTTCCAGATTATGCTGGTA	pGD-C-HA

852/HindIII/3xHA/BglII/R	GATCTACCAGCATAATCTGGAACATCGTATGGATAGCCAGCGTAATCAGGAAC ATCATAAGGGTAACCAGCATAATCTGGAACATCGTATGGATAA	
147/NbEF1A/F	AGCTTTACCTCCCAAGTCATC	qPCR reference
148/NbEF1A/R	AGAACGCCTGTCAATCTTGG	
2904/SoSGV/F	TCGGACCTGGATTGCAGAGG	qPCR
2905/SoSGV/R	AAGATGGGAGCCCTCATCTCC	
